# Time to pay attention to sleep for Alzheimer’s disease in women

**DOI:** 10.1016/j.tjpad.2026.100624

**Published:** 2026-06-12

**Authors:** Ece Bayram

**Affiliations:** Department of Neurology, University of Colorado Anschutz, Aurora, CO, United States

**Keywords:** Sleep, Memory, Tau, Women, Genetic, Alzheimer’s disease

The first Alzheimer’s disease (AD) case described by Alois Alzheimer was a woman suffering from progressive sleep and memory disturbance, paranoia, aggression, and confusion, with plaques and neurofibrillary tangles in brain histology [1]. More than 100 years later, we continue to see AD as a woman-predominant disease defined by cognitive decline including memory deficits with amyloid β plaques and neurofibrillary tangles [2]. Sleep disturbance, however, is left out of the AD diagnostic criteria due to non-specificity, as it increases risk for various neurodegenerative diseases beyond just AD. Nonetheless, research suggests sleep disturbance is still common in AD with a bidirectional relationship [3]. Amyloid β and tau depositions disrupt sleep, and sleep disturbances play a causal role in AD through neuroinflammation, oxidative stress, and glymphatic dysfunction [3]. This causal role puts sleep on the list of potential targets for AD prevention that should be urgently addressed.

The 2024 Lancet Commission on Dementia reported that modifiable risk factors can prevent or delay nearly half of dementia cases, with AD contributing to 70–80% of dementia cases [4]. While sleep was discussed as a potential risk factor, it was ultimately not included as a risk factor due to limited research. Additionally, sleep disturbances are underdiagnosed and undertreated in women, who have the higher risk for AD. Recognizing this gap for sleep in AD literature, Lui and colleagues limited their cohort to only women with prodromal AD to investigate if sleep correlates with memory and tau burden [5]. They also considered the impact of genetic risk levels based on previous research suggesting a role for *APOEe4* in the bidirectional relationship of sleep and tau pathology [3].

Using cross-sectional data for women aged 65 and older with mild cognitive impairment and above average AD genetic risk, Lui and colleagues stratified their cohort into higher and lower AD genetic risk groups based on a cut-off of 75th percentile of polygenic hazard score. Measures included Pittsburgh Sleep Quality Index (PSQI) for self-report sleep quality, Brief Visuospatial Memory Test-Revised for visual memory, Rey Auditory Verbal Learning Test for verbal memory, and composite tau PET uptake within three Braak regions (I/II: entorhinal cortex and hippocampus, III/IV: limbic regions, V/VI: neocortex). Although their sample size was relatively small with 22 women in the higher and 41 women in the lower AD genetic risk group for tau PET analyses, they reported several significant findings with moderate to large effect sizes. Worse sleep was associated with visual memory and more limbic tau burden. There were no sleep associations for verbal memory or tau burden in other Braak regions. Notably, the significant associations were specific to the higher AD genetic risk group. Similar findings were also observed for *APOEe4* carriers specifically. This was not surprising as *APOEe4* is the dominant genetic driver in AD and majority of higher AD genetic risk group in their study had *APOEe4*.

PSQI is a commonly used and validated self-report sleep questionnaire. However, it only measures average sleep patterns over a one-month period. It is susceptible to recall bias, and does not capture daily variations, specific sleep disorders, or underlying processes for sleep disturbance. It is challenging to make strong claims of sleep being a target for AD prevention given the use of this self-report sleep measure in a cross-sectional study with a small cohort of highly educated women mostly identifying as non-Hispanic White. Nevertheless, their findings still link sleep disruption to clinical and biological changes in women with prodromal AD, which prompts further investigation. In fact, as sleep disorders are overlooked in women, subjective measures can be even more helpful for women. On the other hand, several sleep disorders associated with neurodegenerative diseases (e.g., sleep apnea, rapid eye movement sleep behavior disorder) can go unnoticed by the individual and require objective testing. Combined use of subjective and objective measures can be more effective to diagnose women with sleep disturbances. More importantly, when diagnosed, sleep disturbances in women should be treated appropriately as it might protect them against AD.

Another point from this study is the cruciality of stratification in AD. Similar to how anti-amyloid therapies consider *APOEe4*, amyloid positivity and sex to maximize benefit and minimize risks, treating all AD cases as homogeneous can result in misleading findings. Lui and colleagues underscore this by showing that sleep associations were specific to women with higher AD genetic risk level. Additionally, they show that considering the prevalence of a risk factor is not enough. How the factor causes AD may also look different for people based on their genetic risk level, as they found sleep associations with visual memory and limbic tau specifically in the higher AD genetic risk level group, despite this group also having fewer sleep complaints. Thus, including sex or genetic risk level only as covariates to control for in statistical models can result in inaccurate findings. This type of approach obscures effects that differ by sexes/genetic risk levels or are subgroup specific in AD.

The significant sleep association with visual memory, but not verbal memory is also intriguing. Lui and colleagues suggest this might be due to the female verbal advantage prior to dementia onset in AD. Visual and verbal memory are not identical cognitive functions. Their findings make a compelling case to consider visual memory in addition to the commonly used verbal memory measures in AD research. Sleep associations being limited to limbic tau burden is also worth further investigation. This can be due to the bidirectional relationship between tau and sleep as limbic regions play a role in sleep regulation, although the direction of causality cannot be reliably determined from this cross-sectional study.

Dementia, including AD, has been recognized as a public health priority by the World Health Organization since 2012, given its rapidly growing and high prevalence and burden on a global scale. Since then, we have continued to make significant improvements in identifying people at an early stage to develop disease modifying strategies alongside exciting advances with anti-amyloid therapies. In this era of precision medicine, it is clear that “one size fits all” does not work for AD diagnosis or treatment. We are long overdue to develop sex-aware approaches and address the needs of women with AD who remain underrepresented in research, despite making up majority of the AD population. Lui and colleagues suggest sleep as a potential prevention target in older women with higher AD genetic risk, which requires a deeper assessment in longitudinal studies with larger and more diverse cohorts to start providing effective treatment options for women. After all, “Prevention is better than cure” as Desiderius Erasmus said.

## Funding

This work was supported by the 10.13039/100000049National Institute on Aging (R00AG073453).

## Declaration of generative AI and AI-assisted technologies in the manuscript preparation process

There was no use of generative AI or AI-assisted technologies in scientific writing or in figures, images and artwork.

## Declaration of competing interest

The author declares that they have no known competing financial interests or personal relationships that could have appeared to influence the work reported in this paper.
